# Quick SOFA vs Rockall preendoscopy scores for risk assessment in patients with nonvariceal upper gastrointestinal bleeding: a retrospective cohort study

**DOI:** 10.1186/s12245-019-0229-8

**Published:** 2019-03-25

**Authors:** Vladimir Bagin, Evgenii Tarasov, Maria Astafyeva, Evgenii Nishnevich, Vladimir Rudnov, Mikhail Prudkov

**Affiliations:** 1 Department of Anesthesiology and Intensive Care, Municipal Autonomic Health Care Institution, City Clinical Hospital, No. 40, Volgogradskaya 189, Yekaterinburg, Russian Federation 620102; 2 Department of Surgery, Municipal Autonomic Health Care Institution, City Clinical Hospital, No. 40, Volgogradskaya 189, Yekaterinburg, Russian Federation 620102; 3 State Educational Government-Financed Institution of Higher Professional Education, Ural State Medical University, Ministry of Healthcare of the Russian Federation, Repina 3, Yekaterinburg, Russian Federation 620028

**Keywords:** qSOFA score, Rockall score, Prediction scores, Nonvariceal upper gastrointestinal bleeding, NVUGB, Mortality

## Abstract

**Background:**

Several scoring systems are used to evaluate the severity of nonvariceal upper gastrointestinal bleeding (NVUGB) and the risk of rebleeding or death. The most commonly used scoring systems include the Rockall score, Glasgow-Blatchford score, and Forrest classification. However, the use of simpler definitions, such as the quick Sequential Organ Failure Assessment (qSOFA) score, to make a clinical decision is reasonable in areas with limited time and/or material resources and in low- and middle-income countries.

**Methods:**

Patients with NVUGB whose medical records included information required to calculate the qSOFA and Rockall preendoscopy scores at the time of bleeding in the emergency department or another non-intensive care unit department were included in the study. The area under the receiver operating characteristic curve (AUROC) and 95% confidence interval (95% CI) were estimated for the ability of the qSOFA and Rockall preendoscopy scores to predict mortality.

**Results:**

The qSOFA and Rockall preendoscopic scores at the time of bleeding confirmation could be calculated for 218 patients. The mortality rate increased from 3.4% in patients with a qSOFA score = 0 to 88.9% in patients with a qSOFA score = 3 (*P* < 0.001). The AUROC for prediction of mortality was 0.836 (95% CI 0.748–0.924) for the qSOFA score and 0.923 (95% CI 0.884–0.981) for the Rockall preendocopy score (*P* = 0.059).

**Conclusions:**

An increase in the qSOFA score is associated with adverse outcomes in patients with NVUGB. The simple qSOFA score can be used to predict mortality in patients with NVUGB as an alternative when Rockall preendoscopy score is incomplete for which the comorbidity is unknown.

## Background

Nonvariceal upper gastrointestinal bleeding (NVUGB) is a potentially life-threatening disease that requires urgent intervention. Multiple scoring systems are used to evaluate the disease severity and risk of rebleeding or death. The most commonly used scoring systems are the Rockall score [1], Glasgow-Blatchford score [2], and Forrest classification [3]. The Rockall and Glasgow-Blatchford scores include many clinical and laboratory signs. The Forrest classification allows prediction of the rebleeding risk using endoscopic images isolated from the clinical situation. However, the use of simpler definitions to make clinical decisions is reasonable in areas with limited time and/or material resources and in low- and middle-income countries. Recently, as a result of international collaboration between the North American Society of Critical Care Medicine and the European Society of Intensive Care Medicine, the quick Sequential Organ Failure Assessment (qSOFA) score has been integrated into clinical practice [4, 5]. Three variables independently associated with the risk of death and a length of stay in the intensive care unit (ICU) of more than 3 days were identified by studying data from large patient populations and included a respiratory rate ≥ 22 min^−1^, systolic arterial pressure ≤ 100 mmHg, and alteration of mental status (Glasgow Coma Scale score < 15). The results of several meta-analyses show that the simple-to-use qSOFA score allows identification of infected patients with a higher risk of death outside of the ICU [6–9]. Recent studies showed that the qSOFA could be used to predict mortality in noninfected patients in the emergency department (ED) [10] and trauma patients [11]. Quick assessment of the patient’s condition severity with the qSOFA score can contribute to clinical decision-making by the rapid response team and optimize the administration of time and material resources in urgent situations [12].

The aim of our retrospective study was to define whether the qSOFA score calculated in the ED or another non-ICU department was comparable to the Rockall preendoscopy score for the prediction of adverse outcomes in patients with NVUGB.

## Methods

### Study design

Our study was a retrospective cohort study of patients with NVUGB admitted to the Yekaterinburg City Clinical Hospital No. 40 during the period from 2014 to 2016. Yekaterinburg City Clinical Hospital No. 40 is a major acute university hospital with 1506 beds and approximately 45,000 emergency admissions per year, of which 90–120 admissions are patients with NVUGB. Paper charts are filled out for all admitted patients. All patients admitted to the hospital with NVUGB were included in the initial analysis of our study, but only patients with appropriately filled out medical records, including the information needed to calculate the qSOFA and Rockall preendoscopy scores at the time of bleeding in the ED or another non-ICU department, were included in the analysis. One point was assigned to the patients for the presence of each criterion included in the qSOFA score (respiratory rate ≥ 22 min^−1^, systolic arterial pressure ≤ 100 mmHg, and Glasgow Coma Scale score < 15); thus, the total qSOFA score ranged from 0 to 3 points. The Rockall preendoscopy score ranged from 0 to 7 points (Table 1) [1].

**Table 1:** Rockall preendoscopy score

Variables		Score
Age, years	< 60	0
	60–79	1
	≥ 80	2
Shock	Pulse < 100 bpm, SBP > 100 mmHg	0
	Pulse > 100 bpm, SBP > 100 mmHg	1
	Pulse < 100 bpm, SBP < 100 mmHg	2
Comorbidity	None	0
	IHD, CHF, major morbidity	2
	Renal/hepatic failure	3

*bpm* beats per minute, *SBP* systolic blood pressure, *IHD* ischemic heart disease, *CHF* congestive heart failure

The Charlson comorbidity index was used to evaluate patients’ comorbidities. According to this index, a set number of points is applied for each concomitant disease, and 1 point is applied for every decade of life after 49 years [13]. The NVUGB treatment protocol in the study facility complied with the recommendations of the International Consensus Upper Gastrointestinal Bleeding Conference Group [14]. After an initial assessment of the vital signs, a manual rectal examination was performed and a nasogastric tube was inserted. At the next step, one or two peripheral venous catheters were placed, and infusion therapy using Ringer solution was provided until the clinical condition stabilized. Endoscopic examination of the gastrointestinal tract with a Video Gastrointestinal Scope GIF-XP170N (OLIMPUS®) was performed for every patient included in the study at 30–60 min after confirmation of bleeding in the ED or ICU. The diagnosis was defined according to the 10th revision of the International Statistical Classification of Diseases and Related Health Problems (ICD-10). During the endoscopy procedure, epinephrine injection therapy and argon plasma coagulation were performed if active arterial bleeding/spurting, an oozing hemorrhage (without a visible vessel), or a nonbleeding visible vessel was found. Epinephrine injection therapy and argon plasma coagulation were also performed when a nonbleeding adherent clot with a high risk of rebleeding was observed. In other cases, only a diagnostic endoscopic examination was performed. Other methods of endoscopic bleeding therapy (i.e., sclerosant agents, clip/band ligation, cautery, and thrombin/fibrin cyanoacrylate glue) were not available in our facility at the time of treatment of the patients enrolled in the study (2014–2016). In addition to initial resuscitation and endoscopic therapy, the NVUGB treatment protocol included IV infusion of a proton pump inhibitor with subsequent oral administration, prokinetics administration for improvement of endoscopic visualization, eradication therapy if *Helicobacter pylori* was found and other disease-specific treatment options. For patients without additional risk factors, a hemoglobin level < 7 g/dL was considered an indication for red blood cell (RBC) transfusion. For patients with additional risk factors (refractory shock, ischemic heart disease, or infection complications), a more liberal RBC transfusion strategy was applied. Fresh frozen plasma was used in the following cases: when a patient required more than 3–4 units of RBCs within an hour, as part of a massive transfusion protocol, and for correction of severe coagulopathy. In case of rebleeding, the endoscopic treatment was repeated. In rare cases when endoscopic hemostasis was inadequate, open surgery was performed (gastroduodenostomy with sewing of the bleeding vessel at the bottom of the ulcer or tumor).

### Sample size estimation

The sample size was estimated with the statistical software EZR in R commander version 1.37 [15] according to a previous assessment of mortality rates in patients with different Rockall preendoscopy scores in the original study published by Rockall et al. [1]. The following observations were included in the sample size calculation: the mortality rate was 0.2% in patients with a Rockall preendoscopy score 0 point and 16.7% in patients with Rockall preendoscopy score ≥ 1 point. The ratio between patients groups was 1:5.7. The estimated sample size was 194 patients. The sample size in our study exceeded this number because we included all patients with appropriate and relevant data for the evaluation of the preendoscopic qSOFA and Rockall scores, who were hospitalized in the studied facility during the period from 2014 to 2016.

### Statistical analysis

Categorical variables were presented as numbers and percentages and compared with Fisher’s exact test. Continuous variables were presented as the median with interquartile range (IQR) and compared with the nonparametric Mann-Whitney *U* test. The sensitivity, specificity, positive predictive value (+PV), negative predictive value (−PV), positive likelihood ratio (+LR), and negative likelihood ratio (−LR) with 95% confidence intervals (95% CIs) for the ability of a qSOFA and Rockall preendoscopy scores to predict mortality were calculated. The optimal cutoff points for the scores were estimated with the Youden *J* statistics. The area under the receiver operating characteristic curve (AUROC) with 95% CI was estimated for prediction of mortality by the qSOFA and Rockall preendoscopy scores. A two-tailed *P* value < 0.05 was considered statistically significant. The analysis was performed using the statistical software EZR in R commander version 1.37 [15].

## Results

In total, 312 patients with NVUGB were admitted to Yekaterinburg City Clinical Hospital No. 40 during the period from 2014 to 2016. A total of 31 patients died, for a mortality rate of 9.9%. We excluded patients, whose medical records did not contain information about comorbidity or vital signs (as level of consciousness, respiratory rate, or blood pressure) at the time of bleeding confirmation. Appropriate and relevant data required for the evaluation of the preendoscopic qSOFA and Rockall scores at the time of bleeding confirmation could be calculated for 218 patients. After endoscopy, the following conditions were found to cause NVUGB: peptic gastric/duodenal ulcer (ICD-10 codes K25.4, K26.4) in 79 (36.2%) cases; gastric/duodenal erosion (K25.0, K26.0) in 64 (31.1%) cases; Mallory-Weiss tear (K22.6) in 38 (17.4%) cases; malignancy (C16.0, C16.1, C16.2, C16.3, C16.8) in 20 (9.2%) cases; erosive gastritis (K29.0) in 14 (6.4%) cases, and other (K22.1, E16.4) in 3 (1.4%) cases. A total of 145 (66.5%) patients were the male gender. The median age was 59 (IQR 43–71) years, and the median Charlson comorbidity index was 3 (IQR 1–4). The baseline characteristics did not differ significantly between the groups of patients with different qSOFA scores except for the Charlson comorbidity index, which was higher in patients with a higher qSOFA score (Table 2). The bleeding severity was associated with the qSOFA score. Patients with higher qSOFA scores had significantly lower hemoglobin levels; most of these patients had a hemoglobin level < 7 g/dL and required vasopressors. (Table 2).

**Table 2:** Baseline characteristics of the patients enrolled in the study (*n* = 218)

Characteristics	qSOFA = 0 (*n* = 145)	qSOFA = 1 (*n* = 45)	qSOFA = 2 (*n* = 19)	qSOFA = 3 (*n* = 9)	*P* value^a^
Gender male, *n* (%)	100 (69.0)	28 (62.2)	11 (57.9)	6 (66.7)	0.706
Age, years, median (IQR)	57 (43–70)	60 (46–67)	62 (43–76)	64 (58–67)	0.519
CCI, median (IQR)	3 (1–4)	2 (1–4)	4 (1–5)	4 (3–5)	0.036
Diagnosis					
– Peptic gastric/duodenal ulcer, *n* (%)	50 (34.5)	15 (33.3)	8 (42.1)	6 (66.7)	0.247
– Gastric/duodenal erosion, *n* (%)	42 (28.9)	17 (37.8)	3 (15.8)	2 (22.2)	0.356
– Mallory-Weiss tear, *n* (%)	28 (19.3)	7 (15.6)	3 (15.8)	0 (0.0)	0.647
– Malignancy, *n* (%)	15 (10.3)	1 (2.2)	3 (15.3)	1 (11.1)	0.158
– Erosive gastritis, *n* (%)	8 (5.5)	5 (11.1)	1 (5.3)	0 (0.0)	0.527
– Other, *n* (%)	2 (1.4)	0 (0.0)	1 (5.3)	0 (0.0)	0.432
Hemoglobin level, g/dL, median (IQR)	101.0 (76.0–123.0)	88.0 (75.0–105.0)	70.0 (58.0–76.0)	68.0 (60.0–80.0)	< 0.001
Hemoglobin level < 7 g/dL, *n* (%)	24 (16.6)	8 (17.8)	10 (52.6)	5 (55.6)	< 0.001
Vasopressors, *n* (%)	4 (2.8)	7 (15.6)	7 (36.8)	5 (55.6)	< 0.001
Endoscopic therapy, *n* (%)	35 (24.1)	13 (28.9)	7 (36.8)	2 (22.2)	0.642

*qSOFA* quick Sequential Organ Failure Assessment, *IQR* interquartile range, *CCI* Charlson comorbidity index

^a^*P* value comparing qSOFA = 0, qSOFA = 1, qSOFA = 2, and qSOFA = 3

The qSOFA and Rockall preendoscopic score were associated with the outcome. Patients with a higher qSOFA score required hospitalization in the ICU or open surgery and experienced rebleeding more often. The mortality rate increased from 3.4% in patients with a qSOFA score = 0 to 88.9% in those with a qSOFA score = 3 (*P* < 0.001) (Tables 3 and 4).

**Table 3:** Outcomes of the patients enrolled in the study by qSOFA score (*n* = 218)

Outcomes	qSOFA = 0 (*n* = 145)	qSOFA = 1 (*n* = 45)	qSOFA = 2 (*n* = 19)	qSOFA = 3 (*n* = 9)	*P* value^a^
ICU admission, *n* (%)	51 (35.2)	36 (80.0)	19 (100.0)	9 (100.0)	< 0.001
Rebleeding, *n* (%)	16 (11.0)	11 (24.4)	7 (36.8)	3 (33.3)	0.006
Surgery required, *n* (%)	8 (5.5)	6 (13.3)	6 (31.6)	2 (22.2)	0.002
In-hospital mortality, *n* (%)	5 (3.4)	7 (15.6)	8 (42.1)	8 (88.9)	< 0.001

*qSOFA* quick Sequential Organ Failure Assessment, *ICU* intensive care unit

^a^*P* value comparing qSOFA = 0, qSOFA = 1, qSOFA = 2, and qSOFA = 3

**Table 4:** Outcomes of the patients enrolled in the study by Rockall preendoscopic score (*n* = 218)

Outcomes	Rockall ≤ 3 (*n* = 150)	Rockall = 4 (*n* = 31)	Rockall = 5 (*n* = 23)	Rockall = 6 (*n* = 10)	Rockall = 7 (*n* = 4)	*P* value^a^
ICU admission, *n* (%)	63 (42.0)	18 (58.1)	20 (87.0)	10 (100.0)	4 (100.0)	< 0.001
Rebleeding, *n* (%)	12 (8.0)	9 (29.0)	12 (52.2)	3 (30.0)	1 (25.0)	< 0.001
Surgery required, *n* (%)	6 (4.0)	5 (16.1)	7 (30.4)	3 (30.0)	1 (25.0)	< 0.001
In-hospital mortality, *n* (%)	2 (1.3)	3 (9.7)	11 (47.8)	8 (80.0)	4 (100.0)	< 0.001

*ICU* intensive care unit

^a^*P* value comparing Rockall ≤ 3, Rockall = 4, Rockall = 5, Rockall = 6, and Rockall = 7

**Fig. 1:**
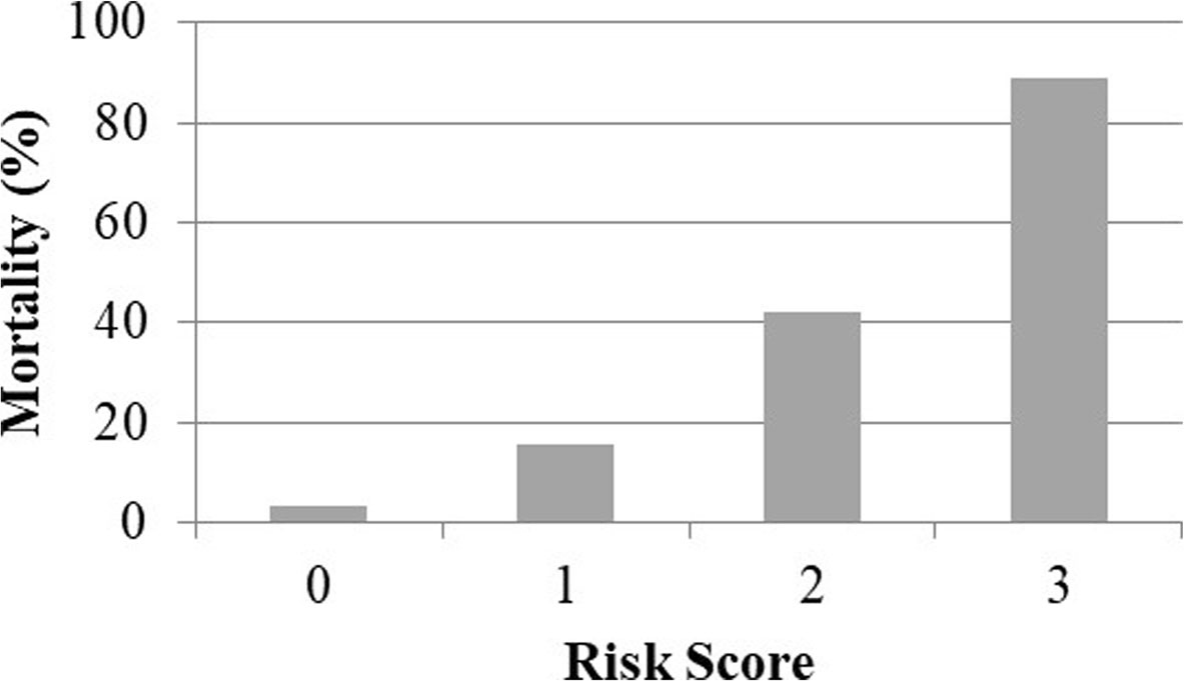
In-hospital mortality by qSOFA score (*n* = 218). The mortality rate was 3.4% in patients with a qSOFA score = 0, 15.6% in those with a qSOFA score = 1, 42.1% in those with a qSOFA score = 2, and 88.9% in those with a qSOFA score = 3 (*P* < 0.001)

**Fig. 2:**
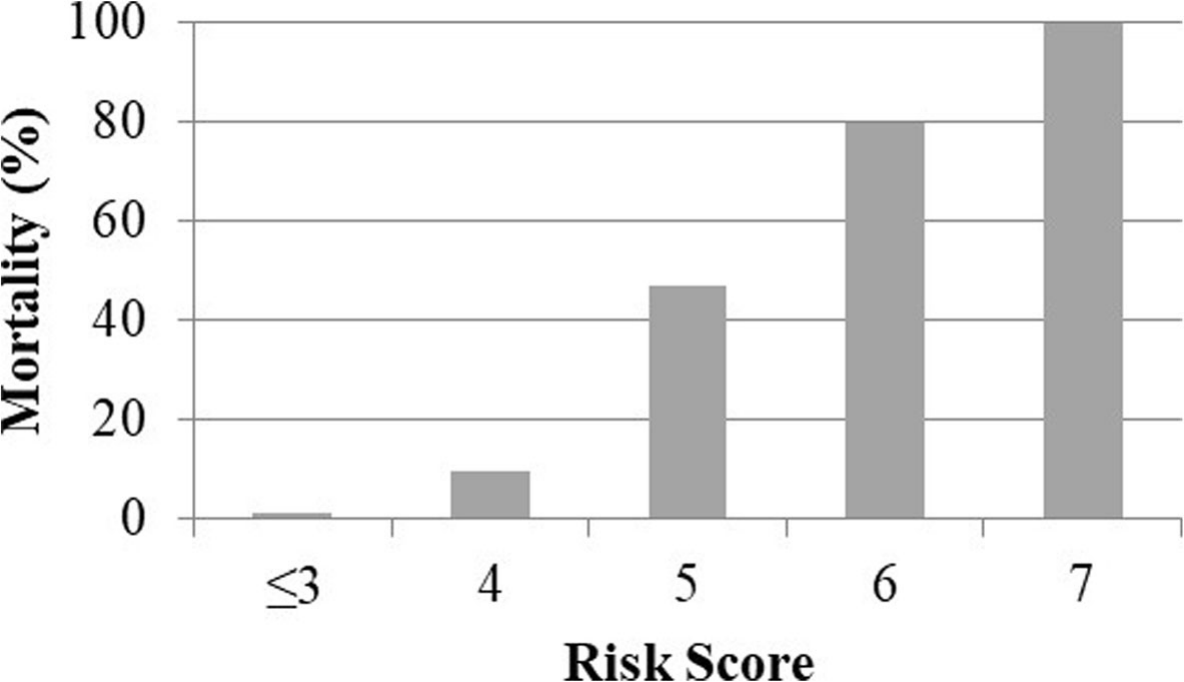
In-hospital mortality by Rockall preendoscopic score (*n* = 218). The mortality rate was 1.3% in patients with a Rockall score ≤ 3, 9.7% in those with a Rockall score = 4, 47.8% in those with a Rockall score = 5, 80.0% in those with a Rockall score = 6, and 100.0% in those with a Rockall score = 7 (*P* < 0.001)

Figures 1 and 2 demonstrate the calibration analysis for the qSOFA and Rockall preendoscopic scores. Mortality is strongly associated with both qSOFA and Rockall preendoscopic scores (Fisher *P* value < 0.001 for both scores).

**Fig. 3:**
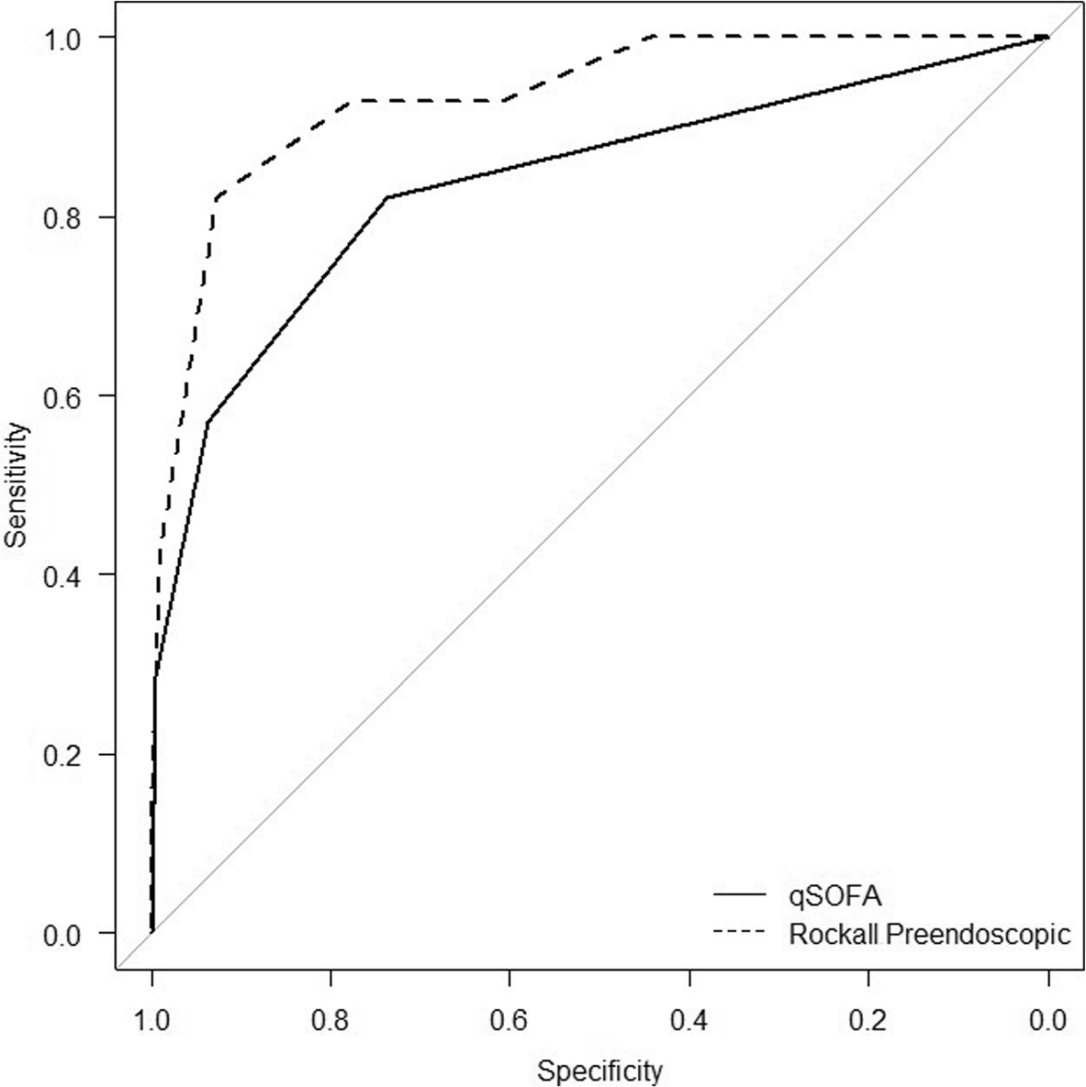
Comparison of the ability of the qSOFA and Rockall preendoscopic scores to predict in-hospital death. qSOFA AUROC = 0.836 (95% CI 0.748–0.924), Rockall AUROC = 0.923 (95% CI 0.884–0.981); *P* = 0.059. qSOFA quick Sequential Organ Failure Assessment, AUROC area under the receiver operating characteristic curve, CI confidence interval

The accuracy of the qSOFA and Rockall preendoscopic scores for prediction of mortality was compared using ROC analysis. Some characteristics (sensitivity, likelihood ratio for negative results, and negative predictive value) for prediction of mortality by the qSOFA score ≥ 1 and Rockall preendoscopic score ≥ 5 did not differ in the study, but the specificity, likelihood ratio for positive result, and positive predictive value were higher for the Rockall score ≥ 5 in comparison with the qSOFA score ≥ 1 (Table 5). For the prediction of mortality, the area under the receiver operating characteristic curve was 0.836 (95% CI 0.748–0.924) for the qSOFA score and 0.923 (95% CI 0.884–0.981) for the Rockall preendocopy score (*P* = 0.059) (Fig. 3).

**Table 5:** Characteristics of the prediction of mortality by the qSOFA and Rockall preendoscopic scores

Score	Se, %, (95% CI)	Sp, %, (95% CI)	+LR (95% CI)	−LR (95% CI)	+PV, %, (95% CI)	−PV, %, (95% CI)	Youden’s index
qSOFA score							
≥ 0	100.0 (87.7–100.0)	0.0 (0.0–1.9)	1.0 (1.0–1.0)	–	12.8 (8.7–18.0)	–	0.000
≥ 1^a^	82.1 (63.1–93.9)	73.7 (66.8–79.8)	3.1 (2.3–4.2)	0.2 (0.1–0.5)	31.5 (21.1–43.4)	96.6 (92.1–98.9)	0.558
≥ 2	57.1 (37.2–75.5)	93.7 (89.2–96.7)	9.1 (4.8–17.1)	0.5 (0.3–0.7)	57.1 (37.2–75.5	93.7 (89.2–96.7)	0.508
3	28.6 (13.2–48.7)	99.5 (97.1–100.0)	54.3 (7.1–417.8)	0.7 (0.6–0.9)	88.9 (51.8–99.7)	90.4 (85.6–94.1)	0.281
Rockall preendoscopic score							
≥ 0	100.0 (87.7–100.0)	0.0 (0.0–1.9)	1.0 (1.0–1.0)	–	12.8 (8.7–18.0)	–	0.000
≥ 1	100.0 (87.7–100.0)	23.7 (17.8–30.4)	1.3 (1.2–1.4)	0.0 (0.0–0.0)	16.2 (11.0–22.5)	100.0 (92.1–100.0)	0.237
≥ 2	100.0 (87.7–100.0)	44.2 (37.0–51.6)	1.8 (1.6–2.0)	0.0 (0.0–0.0)	20.9 (14.4–28.8)	100.0 (95.7–100.0)	0.442
≥ 3	92.9 (76.5–99.1)	61.1 (53.7–68.0)	2.4 (1.9–2.9)	0.12 (0.0–0.4)	26.0 (17.7–35.7)	98.3 (94.0–99.8)	0.540
≥ 4	92.9 (76.5–99.1)	77.9 (71.3–83.6)	4.2 (3.2–5.6)	0.1 (0.0–0.3)	38.2 (26.7–50.8)	98.7 (95.3–99.8)	0.708
≥ 5^a^	82.1 (63.1–93.9)	92.6 (87.9–95.9)	11.2 (6.5–19.0)	0.2 (0.1–0.4)	62.2 (44.8–77.5)	97.2 (93.7–99.1)	0.748
≥ 6	42.9 (24.5–62.8)	99.0 (96.2–99.9)	40.7 (9.6–172.4)	0.6 (0.4–0.8)	85.7 (57.2–98.2)	92.2 (87.6–95.5)	0.419
7	14.3 (4.0–32.7)	100.0 (98.1–100.0)	–	0.9 (0.7–1.0)	100.0 (39.8–100.0)	88.8 (83.8–92.7)	0.143

*qSOFA* quick Sequential Organ Failure Assessment, *Se* sensitivity, *Sp* specificity, *+LR* likelihood ratio for positive results, *−LR* likelihood ratio for negative results, *+PV* positive predictive value, *−PV* negative predictive value, *CI* confidence interval

^a^The optimal cutoff points for the scores were estimated with the Youden *J* statistics

## Discussion

Well-studied scoring systems to predict the prognosis of mortality or rebleeding in patients with NVUGB include the Rockall score [1, 16, 17], Glasgow-Blatchford score [2, 17], and Forrest classification [3]. The Rockall preendoscopic score includes the following clinical signs: age, hemorrhagic shock severity, and concomitant diseases. The Glasgow-Blatchford score includes the hemoglobin level, blood urea level, systolic blood pressure, concomitant diseases, and other clinical signs of bleeding. The Forrest classification is based only on endoscopic signs of bleeding that contribute to the prognosis and treatment course. When faced with limited time and/or resource conditions, a simpler scoring system based only on clinical parameters is a useful approach. The qSOFA score includes the respiratory rate, systolic blood pressure, and level of consciousness. Our study established that the qSOFA score was strongly associated with the mortality rate in patients with NVUBG. Evaluation of the patient’s condition with the qSOFA score can help with management of ED resources, such as prompt transfer of patients to the ICU and/or initiation of early endoscopic treatment. Initially, qSOFA was validated in a cohort of patients with infections [4, 5]. Recently, studies have focused on noninfected patients in the ED [10], trauma patients [11], and patients with liver cirrhosis [18]. We found that the qSOFA score was associated with the bleeding severity, rebleeding frequency, hospitalization in the ICU, requirement for open surgery, and mortality rate (see Tables 2 and 3). The mortality rate was 3.4% in patients with a qSOFA score = 0, 15.6% in those with a qSOFA score = 1, 42.1% in those with a qSOFA score = 2, and 88.9% in those with a qSOFA score = 3 (*P* < 0.001).

The qSOFA and Rockall preendoscopic score AUROCs were compared in our study. For the prediction of mortality, the qSOFA AUROC was 0.836 (95% CI 0.748–0.924) and the Rockall AUROC was 0.923 (95% CI 0.884–0.981); *P* = 0.059 (see Fig. 3). The prognostic prediction of mortality of the Rockall AUROC was higher than that of the qSOFA AUROC, and the *P* value was 0.059 which was slightly greater than the significance level and revealed that the difference between the qSOFA and Rockall score AUROCs was not significant. We defined the optimal cutoff value with the Youden index (*J*) analysis. The optimal cutoff value for the qSOFA score was ≥ 1 point and for the Rockall score was ≥ 5 points. The specificity of the qSOFA score ≥ 1 was lower than of the Rockall preendoscopic score ≥ 5 [73.7 (95% CI 66.8–79.8) vs 92.6% (95% CI 87.9–95.9%)]. The sensitivity of both scoring systems was moderate and comparable: 82.1 (95% CI 63.1–93.9) and 82.1% (95% CI 63.1–93.9%) for the qSOFA score ≥ 1 and Rockall preendoscopic score ≥ 5, respectively. The likelihood ratios for the negative results and the negative predictive values were also comparable between the qSOFA and Rockall preendoscopic scores (see Table 5). In addition to the prognosis of mortality, the qSOFA was useful for prognostic prediction of rebleeding. The rebleeding rate was more than two times higher in patients with a qSOFA score = 1 and more than three times higher in patients with a qSOFA score ≥ 2 compared to those with a qSOFA score = 0 (Table 3). Moreover, patients with a qSOFA score > 0 more often required open surgery because of inadequate endoscopic hemostasis (Table 3). Our study had limitations. First, some nonelectronic medical records for the studied patients did not contain sufficient and relevant information to calculate both the qSOFA and Rockall preendoscopic scores simultaneously at the time of bleeding; thus, these patients were not included in the major analysis. Our study was shifted to more severe cases because 31 (9.9%) of the 312 initially screened patients and 28 (12.8%) of the 218 patients included in the major analysis died. Secondly, we did not perform a comparison of the qSOFA and Rockall preendoscopic scores with scoring models such as the Sequential Organ Failure Assessment Score [19], Acute Physiology and Chronic Health Evaluation Score [20], and others, because the medical records of the patients in the studied facility contained the necessary information only if they were admitted to the ICU during the hospitalization period. Glasgow-Blatchford score [2] is also a well-validated scoring system. However, it is necessary to make a blood urea nitrogen (BUN) test to calculate this score, which in our facility is not an obligatory standard for the treatment of NVUGB and is made only in patients with the high risk of death or comorbidity because of limited material resources. Third, we did not take into account important risk factors, such as administration of nonsteroidal anti-inflammatory drugs, anticoagulants, and other drugs, which could influence the bleeding severity and mortality rate. Fourth, our study is a single-center study performed in a middle-income country with low healthcare costs and thus does not reflect the general population of patients with NVUGB.

## Conclusions

An increase in the qSOFA score is associated with adverse outcomes in patients with NVUGB. The simple-to-use qSOFA score can be used for the prediction of mortality in patients with NVUGB as an alternative when Rockall preendoscopy score is incomplete for which the comorbidity is unknown. A multicenter study is required before making recommendations for use of the qSOFA score in defining the prognosis of patients with NVUGB.

## Abbreviations

−LR: Likelihood ratio for negative results
−PV: Negative predictive value
+LR: Likelihood ratio for positive results
+PV: Positive predictive value
AUROC: Area under the receiver operating characteristic curve
ED: Emergency department
ICD-10: 10th revision of the International Statistical Classification of Diseases and Related Health Problems
ICU: Intensive care unit
IQR: Interquartile range
NVUGB: Nonvariceal upper gastrointestinal bleeding
qSOFA: Quick Sequential Organ Failure Assessment
RBCs: Red blood cells
Se: Sensitivity
Sp: Specificity
